# Prevalence of antibodies to Ro-52 in a serologically defined population of patients with systemic sclerosis

**DOI:** 10.1186/1740-2557-6-2

**Published:** 2009-03-06

**Authors:** Jennifer C Parker, Rufus W Burlingame, Christopher C Bunn

**Affiliations:** 1 Department of Clinical Immunology, Royal Free Hospital, London, NW3 2QG, UK; 2 INOVA Diagnostics Inc., San Diego, California, 92131-1638, USA

## Abstract

**Background:**

Antibodies against Ro-52 have been described in patients with a broad spectrum of autoimmune disease, most commonly in association with anti-Ro-60 in systemic lupus erythematosus and Sjogrens syndrome. However, in inflammatory myositis anti-Ro-52 is frequently present without anti-Ro-60 and is closely linked to the presence of aminoacyl-tRNA synthetase (aats) antibodies. To date there have been no comprehensive reports on the frequency of anti-Ro-52 in systemic sclerosis (SSc), a disease characterised by hallmark autoantibodies that occur in non-overlapping subsets. Clinically, each antibody-defined group has a distinct pattern of organ involvement, some featuring myositis.

**Objectives:**

To determine the frequency of anti-Ro-52 in serologically defined groups of SSc patients and to investigate a possible link with myositis-associated autoantibodies.

**Methods:**

Serum samples from 1010 patients with SSc and 55 and 32 patients with anti-aats and anti-Ku respectively were tested for the presence of anti-Ro-52 using a commercial ELISA.

**Results:**

The prevalence of anti-Ro-52 was 15–38% in nine of the eleven sub-groups. There were no significant differences in mean anti-Ro-52 levels in these groups with the exception of that defined by the presence of anti-U1-RNP. In the remaining groups defined by anti-Ro-60 and anti-aats, anti-Ro-52 was present in 92% and 100% respectively. In sera from non-SSc patients with anti-aats, anti-Ro-52 was detected in 64%.

**Conclusion:**

Anti-Ro-52 is present throughout the SSc population. It is neither more prevalent in the myositis-associated antibody groups nor does it segregate with any other major SSc-specific autoantibodies. The co-existence of anti-Ro-52 with both anti-Ro-60 and anti-aats is confirmed.

## Introduction

Antibodies to the 52 kDa protein Ro-52 were first described in 1988 in addition to antibodies to Ro-60 and La in the serum of patients with Sjogrens syndrome (SS) [1]. Unlike antibodies to Ro-60 and La, they do not produce any specific anti-nuclear antibody staining pattern by indirect immunofluorescence, any precipitin line by immunodiffusion or electroimmunodiffusion, or positive results in enzyme-linked immunosorbent assays (ELISA) containing native antigen [2]. Anti-Ro-52 is mainly detected in the diagnostic laboratory because of the inclusion of recombinant antigen in commercial ELISA and immunoblotting assays [3].

Antibodies to Ro-52 have been shown to be present with anti-Ro-60 (with or without co-existing anti-La) at a high frequency in sera from patients with systemic lupus erythematosus (SLE) and SS [4,5] and one area of interest has centred on their possible pathogenic role in the development of congenital heart block, a complication of the neonatal lupus syndrome [6]. Sera that are monospecific for anti-Ro-52 (ie without anti-Ro-60) have also been described in SS and SLE, but only at a low frequency [2,5]. It has been reported that anti-Ro-52 (mainly monospecific) is present in a large proportion of patients with autoimmune myositis and is closely associated with the myositis-specific anti-aminoacyl-tRNA synthetase (aats) antibodies [7,8]. Neither anti-Ro-60 nor anti-La exhibits this association with anti-aats. This finding has lead to anti-Ro-52 being termed a myositis-associated autoantibody (MAA) [9,10].

SSc is a heterogeneous autoimmune rheumatic disease of unknown aetiology characterised by thickening and fibrosis of the skin and other organs [11]. Virtually all patients have autoantibodies to specific cellular components that are mutually exclusive and correlate with well documented clinical subsets of disease including features of overlap with other connective tissue diseases such as polymyositis (PM) [12]. In this study we have assessed SSc patients characterised for a range of specific antibodies, and additional groups of patients with antibodies to aats and the connective tissue disease associated antibody Ku for the presence of anti-Ro-52. There are currently no reports detailing the frequency of anti-Ro-52 in a cohort of this size.

## Methods

### Patient samples

Serum samples were from 1010 patients diagnosed by an experienced rheumatologist at the Royal Free Hospital, a major tertiary referral centre for SSc, according to the preliminary ACR criteria [13]. All SSc patients had been consented for participation in research studies, approved by the local ethics committee. Antibody groups represented were as follows: anti-centromere (ACA) n = 197, anti-topoisomerase (ATA) n = 210, anti-RNA polymerase III (ARA) n = 207, anti-fibrillarin (AFA) n = 48, anti-Pm-Scl n = 49, anti-U1-RNP n = 58, anti-Ro-60 n = 13, anti-aats (4 anti-Jo-1, 1 anti-PL7, 1 anti-PL12) n = 6, and anti-Ku n = 5. In addition, two further groups were formed from SSc patients with no defined antibody (NDA) n = 173, and those whose sera produced an ANA pattern of fine speckled nucleoplasmic staining with additional homogeneous nucleolar staining (fsnu) n = 44. The latter group represented a heterogeneous population including patients with anti-Th-RNP. All sera were tested for anti-nuclear antibodies by indirect immunofluorescence on HEp-2 cell substrate (Bion inc, Illinois, USA) this was the only method used to define the patients with ACA. The presence of other antibodies was confirmed by counterimmunoelectrophoresis and radioimmunoprecipitation as previously described [14,15]. The antibody frequencies represented in this study do not reflect the prevalence of each antibody type in the SSc patient population as a whole. The samples were selected from a bank of frozen samples stored according to antibody type with patients having no defined specific antibody being over represented.

Following initial analysis a number of sera from non-SSc patients were assessed for presence of anti-Ro-52 to provide additional data for analysis of association with anti-aats and anti-Ku, two under represented antibodies in the selected SSc population. Additional sera tested were from 48 patients with antibodies to aats (36 anti-Jo-1, 8 anti-PL7, 4 anti-PL12) and 32 patients positive for anti-Ku antibodies.

### Anti-Ro-52 ELISA

Anti-Ro-52 was detected by ELISA using purified recombinant Ro-52 (Quanta Lite SS-A 52 ELISA, INOVA Diagnostics, Inc. San Diego, USA) and was performed in accordance with the manufacturer's instructions using the recommended cut-off of 20 U/ml (also shown to be appropriate by local validation (data not shown)).

### Statistical analysis

Statistical analysis was performed using SPSS v 11.0 software. Significant differences in mean anti-Ro-52 levels for the eleven SSc antibody groups, and the extra anti-aats and anti-Ku groups, were determined using one-way analysis of variance (ANOVA) with post-hoc Bonferroni correction. Significant differences were confirmed with the Mann-Whitney U-test. A probability value of < 0.05 was taken to denote statistical significance in all cases.

## Results

Sera from 1010 SSc patients divided into eleven groups based on antibody serology were tested for the presence of anti-Ro-52. The overall frequency of anti-Ro-52 in this selected population was 27% (Table 1).

**Table 1:** Frequency and mean level of anti-Ro-52 in patients tested

**Antibody Group**	**Number of patients**	**Anti-Ro-52 positive (%)**	**Mean anti-Ro52 level (U/ml)**
**ACA**	197	28	22

**AFA**	48	15	18

**ARA**	207	25	22

**ATA**	210	19	22

**fsnu**	44	32	26

**Anti-aats**	6	100	109

**Anti-Ku**	5	60	49

**NDA**	173	28	31

**Anti-Pm-Scl**	49	33	34

**Anti-U1-RNP**	58	38	50

**Anti-Ro-60**	13	92	121

**Total SSc**	1010	27	28

**Anti-aats (non-SSc)**	55	64	78

**Anti-Ku (non-SSc)**	32	28	33

Anti-Ro-52 was present in patients with the major SSc-specific antibodies ACA, ATA and ARA at frequencies of 28%, 19% and 25% respectively with all three groups having a mean anti-Ro-52 level of 22 U/ml. In smaller groups including the minor SSc-specific antibodies AFA, anti-Pm-Scl and the less specific anti-U1-RNP, anti-Ro-52 was present at 15%, 33% and 38%. Two further heterogeneous groups, those without any of the above antibodies but producing a speckled nucleoplasmic and nucleolar ANA pattern and known to contain a percentage of anti-Th-RNP (fsnu) and, with respect to the RFH cohort as a whole, a disproportionately large group of patients with none of the above antibodies or any unifying ANA pattern (NDA) were found to have frequencies of anti-Ro-52 of 32% and 28% respectively. Finally in the three small categories the results were as follows: 13 patients constituted a group where the only identified antibody was anti-Ro-60, 12/13 (92%) were positive for anti-Ro-52. Anti-Ro-60 was also present in a further 19 patients included in other serological groups because of the presence of a more specific antibody. As with those forming the anti-Ro-60 group, 92% of these were positive for anti-Ro-52. Subsequent statistical analysis of the remaining ten groups was not significantly altered by the inclusion or exclusion of these additional nineteen patients. Sera from six SSc patients positive for anti-aats were all positive for anti-Ro-52 and three out of five anti-Ku positive sera were positive for anti-Ro-52.

**Figure 1:**
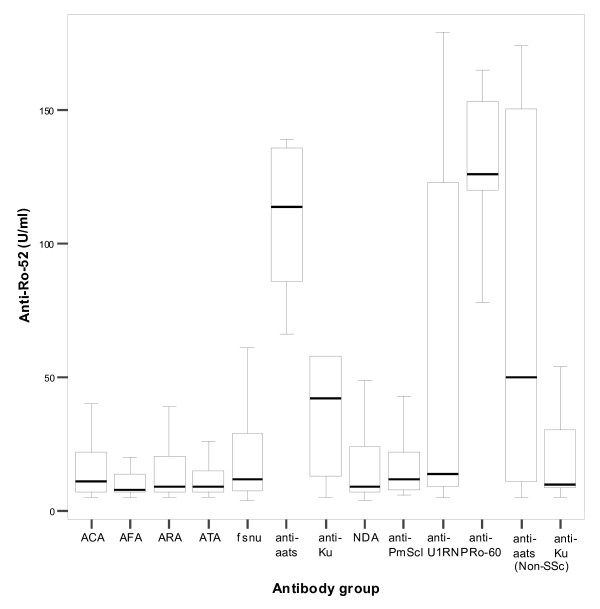
**Anti-Ro-52 levels in 11 serologically defined groups of SSc patients and 2 additional non-SSc groups (anti-aats and anti-Ku antibodies)**. Boxes show interquartile ranges, lines within the boxes indicate median values and lines outside the boxes indicate maximum and minimum values excluding outliers. (See table 1 for numbers of patients in each group).

Comparisons of mean anti-Ro-52 values showed the anti-Ro-60 group had significantly higher levels than all other antibody groups except anti-aats – this small group had significantly higher anti-Ro-52 concentrations than all the remaining groups except anti-Ku. There were no significant differences in anti-Ro-52 levels for the rest of the groups (figure 1) apart from with the anti-U1-RNP group which had significantly different levels to the ACA, ATA, ARA, AFA and NDA groups but not the anti-Pm-Scl, anti-Ku and fsnu groups.

To further investigate the association of anti-Ro-52 with the small anti-aats and anti-Ku groups we tested sera from an additional 48 patients positive for anti-aats and a further 32 sera from anti-Ku positive patients, none of whom were classified as having SSc. Sixty four percent of the anti-aats sera and 28% of those with anti-Ku were positive for anti-Ro-52 (Table 1). The mean levels of anti-Ro52 in the anti-aats patients remained significantly higher than those seen overall in the SSc patient groups whereas the anti-Ku association with anti-Ro-52 was indistinguishable from the SSc groups (with the exception of the anti-Ro-60 and anti-aats groups).

## Discussion

The majority of work to elucidate clinical correlations of anti-Ro-52 has focused on patients with SLE, SS and myositis. Similarly, associations with anti-Ro-60 and anti-aats have been well documented. However, there have been relatively few studies addressing the frequency of this antibody in SSc and no comprehensive reports assessing associations of anti-Ro-52 with the specific autoantibodies that are characteristic of SSc. Initially it was suggested that there was no reactivity of sera from SSc patients with recombinant anti-Ro-52 [16]. Subsequently, reports on the presence and frequency of anti-Ro-52 have provided conflicting results [2,5,8,17,18]. In this study we analysed sera from a substantial cohort of SSc patients representing the entire spectrum of disease, sub-divided into 11 groups by autoantibody profile. Anti-Ro-52 was found to be present at a frequency of at least 15% in all antibody groups tested. The overall frequency of anti-Ro-52 appears to be greater than previously described [8,18].

Consistent with previous observations, anti-Ro-52 was present at high frequency in the group of 13 patients in whom the only identified antibody was anti-Ro-60 [5]. The co-existence of anti-Ro-52 with the myositis-specific aats antibodies was also confirmed. We were able to extend the initial data from a small number of SSc patients by testing additional sera positive for anti-aats and demonstrating a high degree of association with anti-Ro-52, the results being comparable to previous reports [7-9]. As with anti-Ro-60 the reason for the high frequency of anti-Ro-52 in anti-aats positive sera is unknown but it is not due to cross-reactivity between anti-Jo-1 and Ro-52 [7].

In the nine other groups of SSc patients the frequency of anti-Ro-52 varied from 15% in the AFA group to 38% in the anti-U1-RNP group. When detected by traditional methods, as described in this study, it is a characteristic feature of SSc that each patient will typically have only one of a series of hallmark autoantibodies that therefore occur in a mutually exclusive fashion. However, a recent study employing an addressable laser bead immunoassay to analyse autoantibodies in the sera of patients with autoimmune myositis highlighted unusual antibody combinations not previously described [19]. Eventually the wider application of sensitive multiplex methods could confirm whether there is an, as yet, undescribed overlap between the antibody populations. Currently each antibody has been shown to be associated with a disease phenotype and can predict features such as age of onset, extent of skin, and type of organ involvement. The major SSc-specific autoantibodies ACA, ATA and ARA are predictive of the clinical manifestations of limited disease with minimal internal organ involvement, pulmonary fibrosis and renal involvement respectively. As there is no significant difference in the level and frequency of anti-Ro-52 in these three groups it is clear that there is neither an association with any of these antibodies nor, by inference, the clinical outcomes they predict.

As a consequence of the association of anti-Ro-52 with anti-aats and other studies that link myositis-specific anti-Mi-2 and anti-signal recognition particle (SRP) to anti-Ro-52, [9] it was hypothesised that the three antibody groups described as having an increased incidence of muscle involvement (AFA, anti-Pm-Scl, anti-U1-RNP) might show an increased association of anti-Ro-52 compared to the major SSc-specific antibody groups (ACA, ATA, ARA) where myositis is not a prominent feature [12]. The results from this study do not support this proposal.

Anti-Pm-Scl is associated with an overlap syndrome in which up to 80% of SSc patients have inflammatory muscle disease [20]. We did not find any significant difference between the anti-Ro-52 levels in anti-Pm-Scl positive patients and the major SSc antibodies. However, Frank et al reported that the response to Ro-52 was clearly present in anti-Pm-Scl patients while it was rare in ACA and ATA positive SSc patients. It was suggested that detection of anti-Ro-52 may prove useful in discriminating between patients in these antibody groups [8]. This view is not upheld by our findings.

This study also expands the limited data available on anti-Ro-52 in AFA positive patients. Myositis has been shown to be a prominent feature of patients with this antibody. Keonig *et al *detected anti-Ro-52 in six of fourteen autoimmune myositis patients with AFA and in a previous study from this centre 50% of SSc patients with AFA were shown to have myositis [19,21]. There was no association of anti-Ro-52 with AFA, indeed the mean anti-Ro-52 level in the AFA group was lower than in the non-myositis-associated antibody groups.

The results obtained for anti-U1-RNP show a bimodal distribution for anti-Ro-52 with a number of patients having extremely high levels of the antibody. This is likely to account for the higher mean anti-Ro-52 value observed for the anti-U1-RNP group and the subsequent significant differences from the majority of the SSc antibodies not seen for any other antibody group except anti-Ro-60 or anti-aats. We were unable to determine any specific reason to explain this interesting finding.

Only five anti-Ku positive patients were identified in the SSc population and three were positive for anti-Ro-52. Anti-Ku was originally described in Japanese patients with SSc/PM overlap, but in the North American population it appears to be present in a more SLE-like disease [22] The anti-Ku positive patients had higher anti-Ro-52 values than the majority of the other SSc antibody groups (figure 1) but when an expanded number of non-SSc anti-Ku positive sera were analysed there was no significant difference in anti-Ro-52 levels to the SSc population (with the exception of the anti-aats and anti-Ro-60 groups).

Anti-Ro-52 was also present in the two miscellaneous groups. The NDA and fsnu groups in this study were over represented compared to the Royal Free Hospital cohort overall as it was felt that these were possible candidate patients for the presence of anti-Ro-52, there being no other defining antibody detected. The fsnu group was known to contain a proportion of anti-Th-RNP positive patients but due to inconsistent detection by radioimmunoprecipitation, this could not be classed as a homogeneous population. Anti-Ro-52 was detected in 28% and 32% respectively and these values were not statistically different from any other group (except anti-Ro-60 and anti-aats). Of note anti-Ro-52 was the only detectable antibody in 48 patients in the NDA group.

## Conclusion

The data presented here demonstrate that anti-Ro-52 is prevalent throughout the SSc population and does not segregate with any of the major SSc-specific autoantibodies. We confirm the association with both anti-Ro60 and aats antibodies but find no evidence that anti-Ro-52 is found at a higher frequency in SSc patients with myositis-associated antibodies. These findings support the hypothesis that anti-Ro-52 is a general serum marker with limited linkage to a myositis phenotype or other clinical manifestations of SSc.

## Competing interests

The authors declare that they have no competing interests.

## Authors' contributions

CB and RB designed and coordinated the study. JP performed the assays, the statistical analysis and drafted the manuscript. All authors contributed to the final revision of the manuscript.
